# Evolutionary constraints over microsatellite abundance in larger mammals as a potential mechanism against carcinogenic burden

**DOI:** 10.1038/srep25246

**Published:** 2016-04-29

**Authors:** Jung Youn Park, Yong-Rock An, Chul-Min An, Jung-Ha Kang, Eun Mi Kim, Heebal Kim, Seoae Cho, Jaemin Kim

**Affiliations:** 1 Biotechnology Research Division, National Fisheries Research & Development Institute, Gijang gun, Busan, 619-705, Republic of Korea; 2 Cetacean Research Institute, National Fisheries Research & Development Institute, Nam-gu, Ulsan 680-050, Republic of Korea; 3 Department of Agricultural Biotechnology, Seoul National University, Seoul 151-921, Republic of Korea; 4 Interdisciplinary Program in Bioinformatics, Seoul National University, Seoul 151-742, Republic of Korea; 5 C&K Genomics, Seoul National University Research Park, Seoul 151-919, Republic of Korea; 6 National Human Genome Research Institute, National Institutes of Health, Bethesda MD 20892, USA

## Abstract

Larger organisms tend to live longer, have more potentially carcinogenic cells, and undergo more cell divisions. While one might intuitively expect cancer incidence to scale with body size, this assertion does not hold over the range of different mammals. Explaining this lack of correlation, so-called ‘Peto’s paradox’ can likely increase our understanding of how cancer defense mechanisms are shaped by natural selection. Here, we study the occurrence of microsatellite in mammal genomes and observe that animals with expanded body size restrain the number of microsatellite. To take into account of higher mutation rate in the microsatellite region compared to that of genome, limiting the abundance of somatic mutations might explain how larger organisms could overcome the burden of cancer. These observations may serve as the basis to better understand how evolution has modeled protective mechanisms against cancer development.

Cancer is a unique type of genetic disease in which several sequential mutations are necessary, and each mutation drives a wave of cellular proliferation which in turn leads to gradual increases in tumor size, disorganization and malignancy^1^. As cancer arises through the accumulation of mutations, each proliferating cell is at risk of malignant transformation, assuming all cells have similar chances of mutation^2^. Cancer risk is thus expected to increase with larger bodies and longer lifespan, but there appears to be disconnect between prediction and observation across species, a phenomenon termed Peto’s paradox^3^.

There are many hypotheses but limited research efforts to resolve this paradox. Although large bodies evolved independently, some probable and common mechanisms of the effective cancer suppression in large species include lower somatic mutation rates, redundancy of tumor suppressor genes, lower selective advantage of mutant cells, more efficient immune system, shorter telomeres, and fewer reactive oxygen species due to lower basal metabolic rate^2^. Katzourakis *et al*. also suggested that lower levels of tumorgenic endogenous retroviruses in larger bodied species could be the result of evolution of mechanisms capable of limiting retroviral activity^4^. Recently, Varki & Varki provided several explanations for the reported rarity of carcinomas in captive chimpanzees such as differences in diet, their microbiome, and potential environmental factors^5^. Abegglen *et al*. reported that elephants, compared with human, appeared to have multiple copies of tumor suppressor gene (TP53) and also increased level of apoptotic response after DNA damage, which are potential molecular mechanisms of cancer resistance^6^.

Genomes are scattered with numerous simple repeats, and tandem repeats are iterations of repeat units of any size, from a single base pair to thousands of base pairs. The major types of microsatellites are mono-, di-, tri- and tetranucleotide repeats, but units of five or six nucleotides are also classified as microsatellites^7^. These are among the most variable types of DNA sequence in the genome^8^, and genetic variation at many microsatellite loci is characterized by high heterozygosity and the presence of multiple alleles^7^. Notably, the vast number of mutations in cancer cells were directly associated with changes in microsatellites in tumor DNA^9^. The cancer patients harbor mutations in mismatch repair genes^10^,^11^, which leads to failure to correct slippage errors made by DNA polymerases and consequently to give rise to the length changes, microsatellites instability^7^. It seems evident that repetitive elements are “hot spots” for mutagenesis and may serve as markers for detecting other types of mutations throughout the genome^9^,^12^.

In this sense, relating two seemingly disparate contexts, Peto’s paradox and microsatellites across species, may lead to conceptual advances in understanding the mechanisms underlying the animals that have been evolving mechanisms to suppress cancer ever since the origin of multicellularity. In the light of comparative oncology, we explore the hypothesis that differences in microsatellite occurrence across mammalian species have been shaped by natural selection, with larger animals expected to have smaller number of microsatellites in the genome.

## Results and Discussion

We investigated the genome-wide microsatellites (defined as di-, tri-, tetra-, penta-, hexa-nucleotide repeats) across 31 mammalian species (Supplementary Table S1) using RepeatMasker^13^. It is previously suggested that abundance of microsatellite tends to positively correlate with genome size among a variety of eukaryotes, whereas occurrence of microsatellite is negatively correlated with genome size in plants^7^,^14^,^15^,^16^. In mammals in particular, it was evident that the total number of microsatellite does not correlate with genome size (P-value = 0.13) (Supplementary Fig. S1).

**Figure 1:**
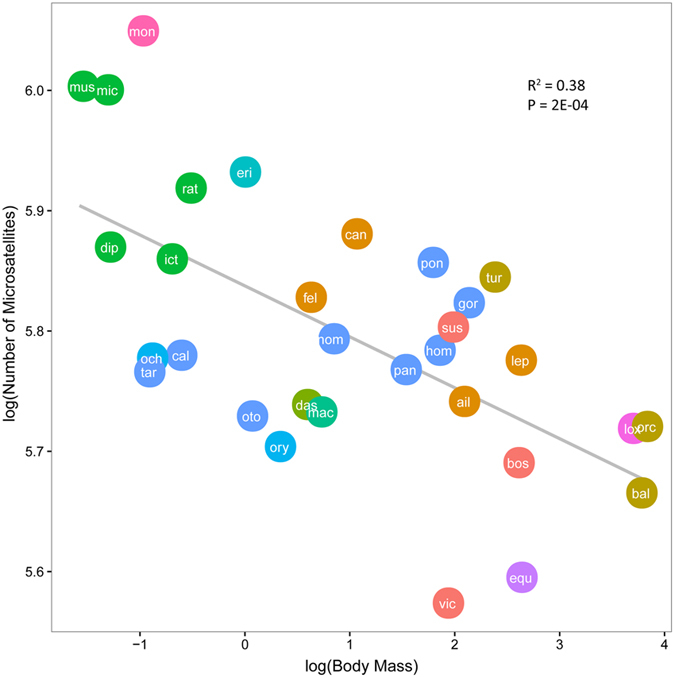
Number of microsatellites in genome against body mass in 31 mammalian species. Both traits are log-transformed, and different colors denote different orders.

Microsatellites can be found anywhere in the genome, both in protein-coding and noncoding regions. Due to their high mutability, microsatellites are thought to play a major role in genome evolution by creating and maintaining quantitative genetic variation^14^,^17^. To understand the selective landscapes in which species evolved in terms of occurrence of microsatellite, we used linear regression to test association between number of microsatellites and body mass. As our surrogate measure of relative level of total number of cells present in each organism, we followed previous studies in the use of body mass^4^,^18^. We observed a significant negative correlation (slope = −0.042, P-value = 2.0E-04 and R^2^ = 0.36), indicating that the number of microsatellites in the whole genome is smaller in species with larger body size (Fig. 1 and Table 1). As multicellular organism expanded the body size, the challenge of suppressing somatic evolution dramatically increases; however, that challenge was successfully tackled in terms of abundance of microsatellite, in which mutation rate is higher than in genome.

As life history traits are often correlated each other, it remains possible that the apparent correlation of body mass with microsatellite occurrence could be confounded by other life history traits. There is increasing evidence that variations in rates of nucleotide substitution show relationships with body size^18^,^19^,^20^ and metabolic rate^17^. Higher metabolic rates, associated with reactive oxygen species (ROS) and metabolic stress along with other by-products of metabolism, can lead to tumorigenesis and appear to be inversely proportional to animal body size^21^. Hence, clarifying if other traits have played a role in determining the number of microsatellites is very crucial in assessing the effect of body mass correctly.

We evaluated the correlation between microsatellite abundance and life history traits with multiple regression model to account for their simultaneous contributions while controlling for potential confounders. In addition, as temperature is known to affect metabolic rate^22^, following the previous study, we considered temperature-corrected mass-specific metabolic rate^23^ as a confounding variable in the model. Body size still remained as the only significant variable confirming that it is the most significant predictor of microsatellite density, which in turn indicates that observed correlation between abundance of microsatellite and body mass is robust against variations in temperature and metabolic rate (Table 1). The result was consistent when mass-specific metabolic rate (not temperature-corrected) was included in the model (Supplementary Table S2).

**Figure 2:**
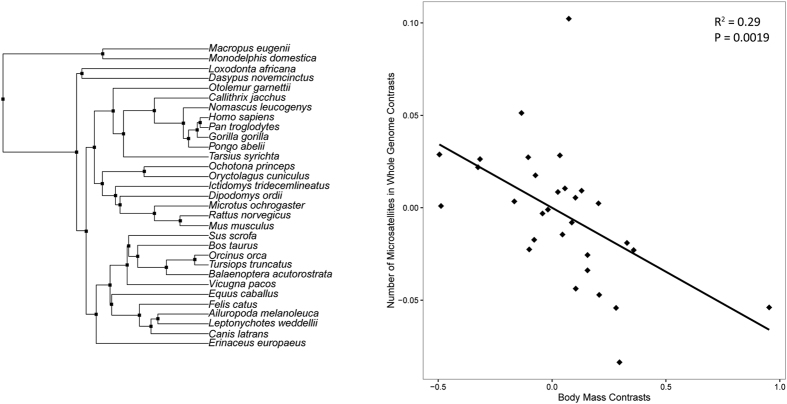
Phylogenetic independent contrasts of body mass versus number of microsatellites in whole genome region. (**a**) Rectangle indicates the contrasts in the phylogeny (Canis familiaris was replaced with Canis latrans in this analysis) (**b**) Relationship between phylogenetically independent contrasts of body mass and contrasts of number of microsatellites in whole genome region.

However, this conclusion still may be premature without phylogenetic comparative analyses of evolution in microsatellites occurrence. When species are used as data points, relationships between raw values of any traits are difficult to interpret, because shared phylogenetic history means that assumptions of statistical independence are likely to be violated^24^,^25^,^26^. It had been demonstrated that such approaches may lead to overestimation, excessively high type I error rates and inaccurate estimations of correlations or slopes^27^,^28^. The correlation was thus re-evaluated in a phylogenetic context. After correcting for phylogenetic proximity, the independent contrasts of body mass versus number of microsatellites were correlated significantly and negatively (slope = −0.069, P-value = 0.0019) (Fig. 2). Although this method is also limited for loss of statistical power and its reliance on the assumption of constant rates of trait evolution through time^29^,^30^, a consistent result supports the evidence that microsatellite abundance is significantly associated with body size.

Comparing genomic regions of interest for cancer research such as proto-oncogenes, tumor suppressor genes or whole protein-coding genes, widespread in mammalian genomes can provide important insights into how these classes of genes have been in subject to natural selection^31^. We first observed that body mass still contributed significantly (P-value < 0.05) to the microsatellite occurrence within genic region under both simple (Supplementary Fig. S2) and multiple regression models (Supplementary Table S3) but not under comparative phylogenetic analysis. As we focus our attention to proto-oncogene and tumor suppressor genes, a negative trend was observed between microsatellite abundance and body mass, but the correlation was not highly significant (slope = −0.059, R^2^ = 0.12, P-value = 0.076) (Supplementary Fig. S3). More complete results can be expected with better quality of genome annotation and better definition of proto-oncogene and tumor suppressor genes across species (for example, different numbers of copies of the genes can also alter the level of cancer resistance^6^,^32^). Interestingly, common minke whale characterized itself as a stricter regulator than any other species. Common minke whale seemed to very extremely suppress the occurrence of microsatellites in genic region, proto-oncogene and tumor suppressor gene regions where the accumulated mutations can cause the cancer developments at relatively higher chance.

The extension of evolutionary thinking into cancer biology has contributed to realization that cancer defenses both between tissues within individual and between species have been influenced by natural selection^33^. Our results indicate that larger mammals tend to exert more effective control over microsatellite occurrence throughout the genome. We suggest that a driving force for this restraint in larger and longer-lived animals is their higher expected cancer rates given the number of cells and number of cell divisions that occur.

## Methods

### Data and identifications of microsatellite

We downloaded 30 complete mammalian genomes from the NCBI and USCS databases and the assembled whole genome of common minke whale^34^ to finally retain 31 species. Microsatellites were identified (masked as simple repeats) using RepeatMasker version 4.0.5 (http://repeatmasker.genome.washington.edu)^13^ with the “no_is” parameter to skip bacterial insertion element check. The human proto-oncogene and tumor suppressor gene were retrieved from the UniprotKB (KW-0656 and KW-0043 respectively)^35^ and found the orthologous genes across 27 publically available species using Ensembl database^36^. We then used BLAST to search the genomic sequence of the orthologous gene for common minke whale to finally retain the lowest common number of 27 gene set throughout 28 mammalian species^37^.

### Multiple linear regression analysis and Phylogenetic comparative analyses

Life history traits correlate with each other, and thus body size could in principle be a surrogate measure of a different life history trait, as has been previous shown for body temperature and metabolic rate. Mammalian life history data (Supplementary Table S1) was mainly taken from PanTHERIA database^38^ and the phylogenetic tree from TimeTree^39^. We used the phylogenetically independent contrasts (PIC) approach as implemented by the Analysis of Phylogenetic and Evolution (APE)^40^ package in R version 3.2.2 (http://cran.r-project.org/) to control for shared ancestry^26^. To correct for the effect of temperature in metabolic rate, mass-specific metabolic rate of each species was transformed to 25 °C, following the previous study^23^. A range of average activation energy (E = 0.4, 0.65 and 0.8) was considered, and the result was robust (Supplementary Tables S2 and S3). The relationship between the standardized independent contrasts were then investigated through ordinary least squares regression analysis, with regression lines constrained to pass through the origin^28^. Traits were log-transformed in all regression analyses. The lm function in R was used to perform regression analyses.

## Additional Information

**How to cite this article**: Park, J. Y. *et al*. Evolutionary constraints over microsatellite abundance in larger mammals as a potential mechanism against carcinogenic burden. *Sci. Rep*. **6**, 25246; doi: 10.1038/srep25246 (2016).

## Supplementary Material

Supplementary Information

## Figures and Tables

**Table 1:** Relationships between number of microsatellites and life history traits in non-phylogenetic models.

Dependent variable	Simple linear regression				Multiple linear regression	
	df^1^	slope(beta)	R^2^	*P-value*	slope (beta)	*P-value*
body mass	29	−0.042	0.38	<0.001	−0.042	0.023
2temperature-corrected mass-specific basal metabolic rate	29	0.047	0.25	<0.01	0.00077	0.97

^1^df denotes degree of freedom

^2^activation energy of E = 0.65 was used to correct for temperature. Results from other E ranges are shown in Supplementary Table S2.
