# Supplementary material for: Evolutionary constraints over microsatellite abundance in larger mammals as a potential mechanism against carcinogenic burden
Source: Sci Rep. 2016 Apr 29;6:25246. doi: 10.1038/srep25246 (PMC4850439; doi:10.1038/srep25246)
Supplement: Supplementary Information [file srep25246-s1.pdf]

Evolutionary constraints over microsatellite abundance in larger mammals as a potential mechanism against carcinogenic burden

Jung Youn Park, Yong-Rock An, Chul-Min An, Jung-Ha Kang, Eun Mi Kim, Heebal Kim, Seoae Cho, Jaemin Kim

SUPPLEMENTARY INFORMATION

1. **Supplementary Figures**

   Supplementary Figure 1………………………………………………….. 2

   Supplementary Figure 2………………………………………………….. 3

   Supplementary Figure 3………………………………………………….. 4

2. **Supplementary Table**

   Supplementary Table 1…………………………………………………… 5

   Supplementary Table 2…………………………………………………… 6

   Supplementary Table 3…………………………………………………… 7

Supplementary Figure S1. Number of microsatellites versus log-transformed genome size of 31 mammalian species.

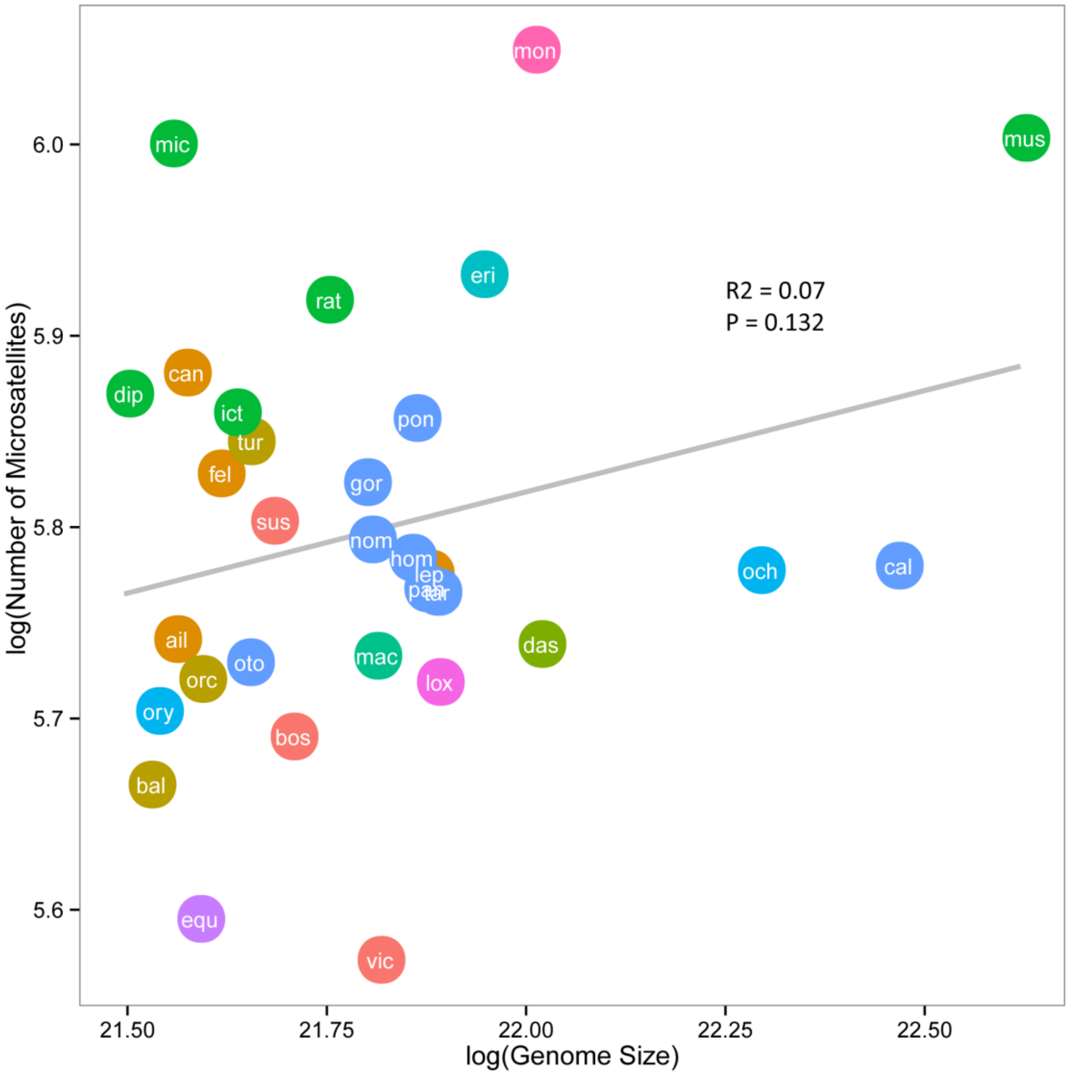

Supplementary Figure S2. Number of microsatellites in genic region versus body mass in 29 mammalian species.

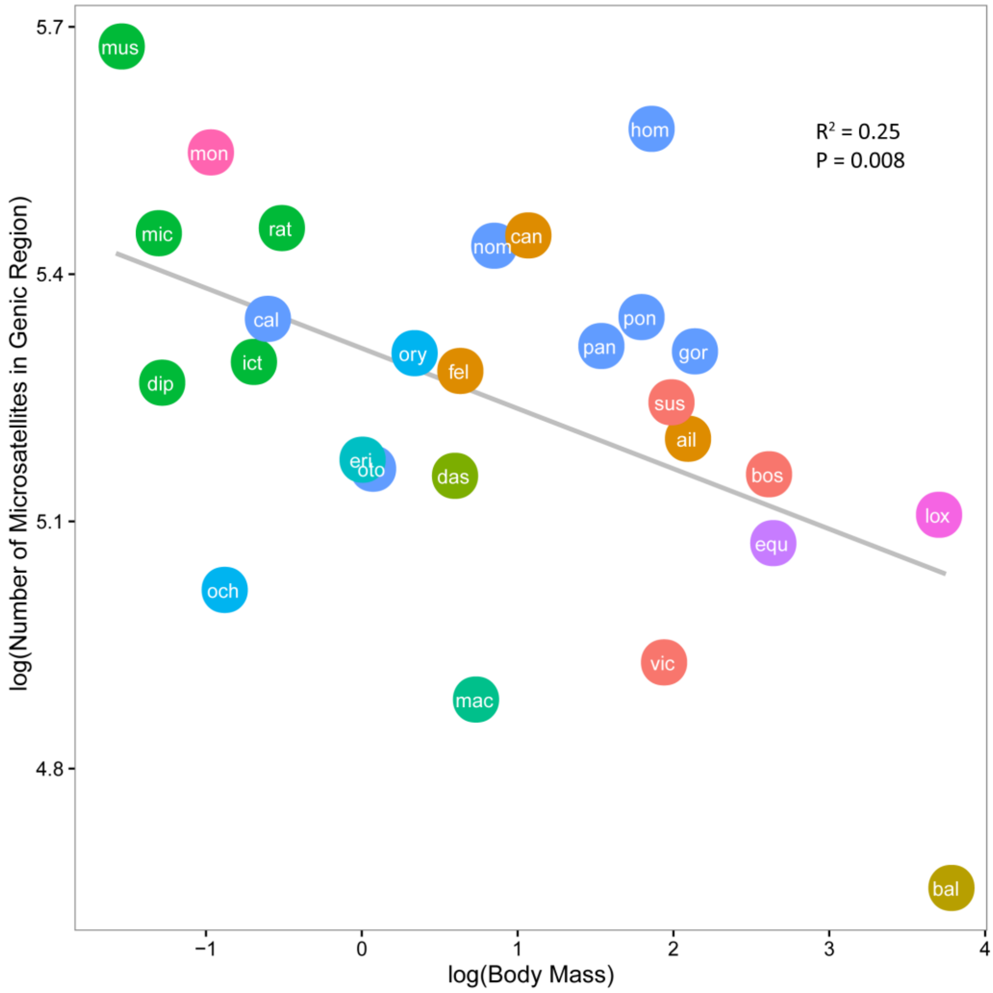

Supplementary Figure S3. Number of microsatellites in oncogene and tumor suppressor gene regions versus body mass in 29 mammalian species.

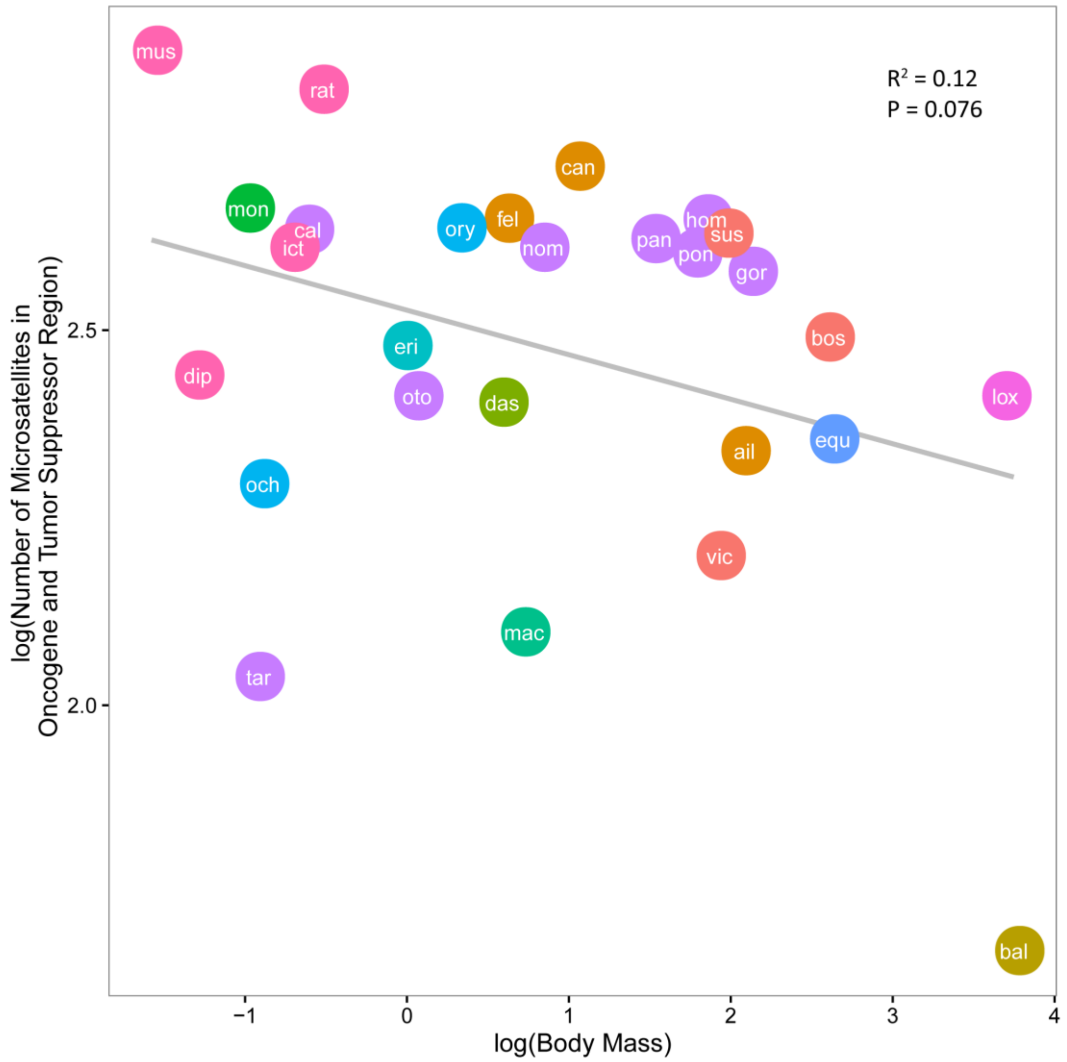

Supplementary Table S1. Summary of mammalian species in this study.

Species	Order	ABB[1]	Microsatellites[2]	BM[3]	BMR[4]	T_b[5]	Ref. BMR	Ref. T_b
Ailuropoda melanoleuca	Carnivora	ail	5.741	114.000	10757.0	38.9	[1]	[2]
Balaenoptera acutorostrata	Cetacea	bal	5.665	5587.094	365737.1	35.3	[3]	[4]
Bos taurus	Artiodactyla	bos	5.690	377.161	33700.4	38.4	[5]	[2]
Callithrix jacchus	Primates	cal	5.780	0.230	152.0	36	[6]	[2]
Canis familiaris	Carnivora	can	5.880	10.770	7467.2	38.4	[5]	[2]
Dasypus novemcinctus	Cingulata	das	5.739	3.643	865.0	34.5	[6]	[5]
Dipodomys ordii	Rodentia	dip	5.869	0.048	62.5	34.6	[5]	[2]
Equus caballus	Perissodactyla	equ	5.595	401.799	62473.0	38.3	[5]	[2]
Erinaceus europaeus	Erinaceomorpha	eri	5.932	0.930	471.7	35.6	[5]	[2]
Felis catus	Carnivora	fel	5.828	3.942	1845.6	38.1	[5]	[2]
Gorilla gorilla	Primates	gor	5.823	126.294	27453.3	35.5	[7]	[2]
Homo sapiens	Primates	hom	5.784	66.548	14107.6	37	[6]	[2]
Ictidomys tridecemlineatus	Rodentia	ict	5.860	0.187	103.7	35.7	[5]	[5]
Leptonychotes weddellii	Carnivora	lep	5.775	395.800	88908.3	37.3	[5]	[2]
Loxodonta africana	Proboscidea	lox	5.719	4652.270	246771.7	36.2	[5]	[2]
Macropus eugenii	Diprotodontia	mac	5.732	4.967	1375.7	36.5	[5]	[2]
Microtus ochrogaster	Rodentia	mic	6.000	0.046	128.6	37.9	[8]	[2]
Monodelphis domestica	Didelphimorphia	mon	6.049	0.099	60.0	32.6	[6]	[2]
Mus musculus	Rodentia	mus	6.003	0.026	57.3	36.7	[5]	[2]
Nomascus leucogenys	Primates	nom	5.793	6.510	181.0	39	[5]	[2]
Ochotona princeps	Lagomorpha	och	5.777	0.121	166.8	40.1	[8]	[2]

Orcinus orca	Cetacea	orc	5.720	6314.379	407850.6	36	[9]	[2]
Oryctolagus cuniculus	Lagomorpha	ory	5.704	2.006	1140.0	39	[6]	[2]
Otolemur garnettii	Primates	oto	5.729	1.086	558.2	36	[5]	[2]
Pan troglodytes	Primates	pan	5.768	31.673	1407.6	35.7	[6]	[2]
Pongo abelii	Primates	pon	5.857	57.348	1514.3	37.1	[5]	[2]
Rattus norvegicus	Rodentia	rat	5.918	0.282	307.5	37.2	[5]	[2]
Sus scrofa	Artiodactyla	sus	5.803	89.243	17581.9	38.8	[6]	[2]
Tarsius syrichta	Primates	tar	5.766	0.114	76.7	33.8	[5]	[2]
Tursiops truncatus	Cetacea	tur	5.845	223.333	55441.1	36.3	[5]	[2]
Vicugna pacos	Artiodactyla	vic	5.574	80.000	27867.7	39.3	[10]	[2]

[1]abbreviations

[2]number of microsatellites (log-transformed)

[3]body mass in kilograms. Body mass estimates were averaged across different studies. [2, 5, 11-13]

[4]basal metabolic rate (mL O2 / h). We used 20.08 Jml^{-1} O_2 to convert oxygen consumption to heat production [14].

[5]body temperature (in Celsius)

Supplementary Table S2. Relationships between number of microsatellites in the whole genome and life history traits in non-phylogenetic models under different assumption on activation energy.

**Dependent variable**	***Simple linear regression***				***Multiple linear regression***	
	df [1]	slope (beta)	R^2	*P-value*	slope (beta)	*P-value*
body mass	29	-0.042	0.38	< 0.001	-0.043	0.019
temperature-corrected mass-specific basal metabolic rate (E=0.4)	29	0.046	0.24	< 0.01	-0.0017	0.94
body mass	-	-	-	-	-0.041	0.025
temperature-corrected mass-specific basal metabolic rate (E=0.8)	29	0.047	0.26	< 0.01	0.0022	0.97
body mass	-	-	-	-	-0.046	0.013
mass-specific basal metabolic rate	29	0.045	0.23	< 0.01	-0.0056	0.81

[1]df denotes degree of freedom

Supplementary Table S3. Relationships between number of microsatellites within genic region and life history traits in non-phylogenetic models under different assumption on activation energy.

**Dependent variable**	***Simple linear regression***				***Multiple linear regression***	
	df [1]	slope (beta)	R^2	*P-value*	slope (beta)	*P-value*
body mass	25	-0.073	0.25	< 0.01	-0.10	0.030
temperature-corrected mass-specific basal metabolic rate (E=0.4)	25	0.060	0.10	0.1	-0.043	0.44
body mass	-	-	-	-	-0.10	0.030
temperature-corrected mass-specific basal metabolic rate (E=0.65)	25	0.059	0.11	0.099	-0.044	0.44
body mass	-	-	-	-	-0.10	0.030
temperature-corrected mass-specific basal metabolic rate (E=0.8)	25	0.059	0.11	0.098	0.043	0.44
body mass	-	-	-	-	-0.10	0.028
mass-specific basal metabolic rate	25	0.060	0.10	0.10	-0.043	0.44

[1]df denotes degree of freedom

Reference

1. Nie Y, Speakman JR, Wu Q, Zhang C, Hu Y, Xia M, Yan L, Hambly C, Wang L, Wei W: **Exceptionally low daily energy expenditure in the bamboo-eating giant panda**. *Science* 2015, **349**(6244):171-174.
2. Amos W, Filipe LN: **Microsatellite frequencies vary with body mass and body temperature in mammals, suggesting correlated variation in mutation rate**. *PeerJ* 2014, **2**:e663.
3. Hind A, Gurney W: **The metabolic cost of swimming in marine homeotherms**. *Journal of Experimental Biology* 1997, **200**(3):531-542.
4. Olsen E, Sunde J: **Age determination of minke whales (Balaenoptera acutorostrata) using the aspartic acid racemization technique**. *Sarsia: North Atlantic Marine Science* 2002, **87**(1):1-8.
5. Sieg AE, O'Connor MP, McNair JN, Grant BW, Agosta SJ, Dunham AE: **Mammalian metabolic allometry: do intraspecific variation, phylogeny, and regression models matter?** *The American Naturalist* 2009, **174**(5):720-733.
6. Tourmente M, Roldan ER: **Mass-Specific Metabolic Rate Influences Sperm Performance through Energy Production in Mammals**. *PloS one* 2015, **10**(9):e0138185.
7. Pontzer H, Raichlen DA, Gordon AD, Schroepfer-Walker KK, Hare B, O'Neill MC, Muldoon KM, Dunsworth HM, Wood BM, Isler K: **Primate energy expenditure and life history**. *Proceedings of the National Academy of Sciences* 2014, **111**(4):1433-1437.
8. Lovegrove BG: **The zoogeography of mammalian basal metabolic rate**. *The American Naturalist* 2000, **156**(2):201-219.
9. Worthy GA, Worthy TA, Yochem PK, Dold C: **Basal metabolism of an adult male killer whale (Orcinus orca)**. *Marine Mammal Science* 2014, **30**(3):1229-1237.
10. Riek A: **Relationship between field metabolic rate and body weight in mammals: effect of the study**. *Journal of Zoology* 2008, **276**(2):187-194.
11. Jones KE, Bielby J, Cardillo M, Fritz SA, O'Dell J, Orme CDL, Safi K, Sechrest W, Boakes EH, Carbone C: **PanTHERIA: a species-level database of life history, ecology, and geography of extant and recently extinct mammals: Ecological Archives E090-184**. *Ecology* 2009, **90**(9):2648-2648.
12. Tacutu R, Craig T, Budovsky A, Wuttke D, Lehmann G, Taranukha D, Costa J, Fraifeld VE, De Magalhães JoP: **Human ageing genomic resources: integrated databases and tools for the biology and genetics of ageing**. *Nucleic acids research* 2012:gks1155.
13. White CR, Seymour RS: **The role of gravity in the evolution of mammalian blood pressure**. *Evolution* 2014, **68**(3):901-908.
14. Schmidt-Nielsen K: **Animal physiology: adaptation and environment**: Cambridge University Press; 1997.
